# Diffangle-Grasp: Dexterous Grasp Synthesis via Fine-Grained Contact Generation and Natural Pose Optimization

**DOI:** 10.3390/biomimetics10080492

**Published:** 2025-07-25

**Authors:** Meng Ning, Chong Deng, Ziheng Zhan, Qianwei Yin, Xue Xia

**Affiliations:** 1 School of Intelligent Manufacturing, Jiangnan University, Wuxi 214122, China; 8201602011@jiangnan.edu.cn (M.N.); 6230805042@stu.jiangnan.edu.cn (C.D.); 6230811019@stu.jiangnan.edu.cn (Q.Y.); 6230805118@stu.jiangnan.edu.cn (X.X.); 2 Jiangsu Key Laboratory of Advanced Food Manufacturing Equipment and Technology, Wuxi 214122, China

**Keywords:** grasp generation, hand–object interaction, CVAE model, diffusion model for shared potential space, natural gesture supervision, human-like grasping

## Abstract

Grasping objects with a high degree of anthropomorphism is a critical component in the field of highly anthropomorphic robotic grasping. However, the accuracy of contact maps and the irrationality of the grasping gesture become challenges for grasp generation. In this paper, we propose a reasonably improved generation scheme, called Diffangle-Grasp, consisting of two parts: contact map generation based on a conditional variational autoencoder (CVAE), sharing the potential space with the diffusion model, and optimized grasping generation, conforming to the physical laws and the natural pose. The experimental findings demonstrate that the proposed method effectively reduces the loss in contact map reconstruction by 9.59% in comparison with the base model. Additionally, it enhances the naturalness by 2.15%, elevates the success rate of grasping by 3.27%, reduces the penetration volume by 11.06%, and maintains the grasping simulation displacement. The comprehensive comparison and qualitative analysis with mainstream schemes also corroborate the rationality of the improvement. In this paper, we provide a comprehensive account of our contributions to enhancing the accuracy of contact maps and the naturalness of grasping gestures. We also offer a detailed technical feasibility analysis for robotic human grasping.

## 1. Introduction

The development of humanoid robotics and virtual technologies has spawned technological innovations and scientific advances in this avant-garde field. Among them, the manipulation capability of a bionic dexterous end-effector is the core medium and key technology for robot interactions with the physical environment [1]. Achieving human-like high-fidelity grasping manipulation is one of the core objectives of current research. However, all high-level research oriented towards functional tasks, such as tool use and fine manipulation, relies on a more fundamental premise: how to generate stable, natural, humanoid, and physically reasonable grasping postures. Researchers have approached this from a theoretical foundation, delving into the core issue of hand–object interaction modeling [2,3,4,5,6,7]. It is worth emphasizing that generating high-quality humanoid grasping poses is a critical and challenging research goal in itself, as well as a necessary foundational step towards dexterous manipulation of actual robots. Current research focuses on modeling and innovative simulation methods for hand–object interaction mechanisms, with the central challenge being to predict the precise pose of a human hand when grasping an object.

In order to surmount the aforementioned challenges, a significant number of scholars have conducted relevant research. A substantial body of research has demonstrated that the generation of typical grasping gestures can be subdivided into two distinct stages [8]. First, a generative model, such as a conditional variational autoencoder (CVAE) [9], is used to predict and generate the contact maps [10] or component maps [11] of the grasping object. Subsequently, the implementation of iterative optimization or reinforcement learning algorithms facilitates the generation of grasping gestures that are both natural and reasonable.

In the field of contact graph generation, Shao et al. [12] proposed the UniGrasp grasp generation method, which uses a multi-stage point set selection network (PSSN) for contact point prediction. Nevertheless, the network architecture is susceptible to cascading error accumulation and is constrained in its capacity to model two-finger and three-finger grasping scenarios. ContactGrasp [13] learns thermodynamic residual features of object surfaces using the ContactDB dataset [14], enabling the generation of high-resolution contact heatmaps. Deep learning-based contact map prediction methods, such as ContactNet [10] and DeepContact [5], establish end-to-end contact generation models. With the development of generative models, Wu et al. [15] innovatively constructed a generative framework based on conditional variational autoencoders (CVAEs), enhancing the rationality of contact maps through physical space projection and a dual-layer optimization mechanism. However, existing methods primarily focus on modeling global contact probabilities of the hand, with limitations in fine-grained contact representation. To address this issue, Liu et al. [11] proposed the ContactGen method, introducing a hierarchical conditional variational autoencoder architecture. Modeling the joint probability distribution of contact positions, hand anatomical regions, and contact directions significantly improves the granularity of contact representation. Zhao et al. [16] proposed GrainGrasp, which breaks away from the whole-hand adjustment paradigm and constructs a modular contact optimization framework based on CVAEs. The independent prediction of the contact maps for each fingertip facilitates precise adjustment of the contact positions, thereby enhancing both the spatial resolution and physical consistency of the contact maps.

However, the existing generative model-based approaches mentioned above mainly rely on a single conditional variational autoencoder (CVAE) to learn the mapping from the object to the contact maps and directly decode the generated contact map in its potential space. These approaches have made significant progress, but the possible space of a single CVAE may result in a loss of features that do not adequately capture the detailed features required for fine-grained contacts.

In the domain of grasping optimization research, the primary objective is to enhance grasping stability and reduce the penetration rate. To this end, researchers predominantly employ two technical approaches: physics-based simulation optimization and force closure analysis. Classic methods like GraspIt [17] use physical simulation to iteratively optimize grasping poses, while force closure methods [18] can ensure static stability but are limited by inherent flaws, like single-mode grasping and improper adjustment of joint angle ranges. With the development of the MANO [19] parametric hand model, Jiang et al. [10] proposed the GraspCVAE framework, innovatively introducing a contact consistency loss function and penetration penalty mechanism to optimize MANO parameters; Zhou’s team [20] proposed the TOCH method, which models the spatiotemporal correspondence between the hand and object to effectively correct non-physically reasonable hand motion trajectories. However, prevailing optimization methodologies continue to confront substantial challenges, particularly in the context of dexterous hand operations, where the optimization of multi-finger cooperative motion necessitates establishment of an accurate joint spatial mapping model. In recent research, Graingrasp [16] proposed the Direction Consistency Optimization (DCoG) method, which achieves independent adjustment of each finger by constructing multiple energy fields, making progress in improving grasping stability and reducing penetration rates. However, due to the absence of an explicit joint angle supervision mechanism, the generated gesture exhibits kinematic singularities and insufficient ergonomic rationality.

**Figure 1:**
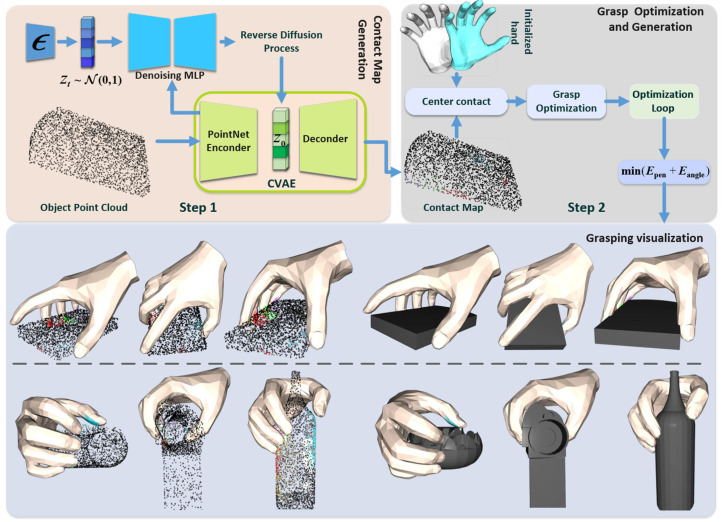
Overview of the Diffangle-Grasp framework. Step 1: Based on the CVAE and diffusion model network in shared potential space, using the input object point cloud, the object grasping contact map is generated through encoder coding, diffusion model inverse iteration, and decoder processing. Step 2: Then, combining multiple physical constraints on the energy term and the joint angle constraints, the final grasping gesture is generated by iteratively optimizing the minimization of the total energy function, and the final grasping gesture is generated. The effect visualization is also generated.

Inspired by the above research, the core objective of this study is to address a fundamental challenge, namely, to construct a physically reasonable and highly human-like natural grasping gesture method that can be utilized to generate object point clouds. This result will provide more reliable basic inputs for subsequent research, such as task-constrained grasping optimization and simulation-to-reality migration, ultimately empowering more robust robotic dexterity. Diffangle-Grasp is proposed for grasp generation, which includes two key components: contact map generation, based on a conditional variational autoencoder (DiffCVAE) that shares the latent space with a diffusion model, and optimized grasp generation, which conforms to physical laws and natural gestures, as shown in Figure 1. Our contributions are summarized as follows:

- We propose an improved contact map graph generation model, DiffCVAE. Unlike existing models that rely on a single model or a simple combination of CAVEs, the core of DiffCVAE is the construction of a shared latent space. The diffusion model is embedded into the potential space of the CVAE to refine the reconstruction of the initial potential variables. The quality of the contact maps is improved by utilizing the iterative refinement capability of the diffusion model on the shared potential space. Meanwhile, compared to the whole hand adjustment, the method can specifically generate fine contact maps for each gesture.
- We propose PNGR, a grasp optimization method that generates intuitive and effective dexterous hand grasps using the aforementioned fine-grained contact maps. By incorporating supervision from physical constraints and natural poses, this approach achieves grasps that are more natural and physically plausible.

By improving the contact map generation network and adjusting the grasping optimization process, the precision of contact map generation is enhanced. Furthermore, the integration of finger angle supervision within the grasping optimization process has been demonstrated to enhance the precision and congruence of dexterous grasping movements with respect to natural gestures. This refinement has been shown to result in an improvement in grasping stability. The ultimate goal of these technological improvements and optimizations is to obtain physically more stable grasps, i.e., reduced penetration volumes and simulated displacements, more natural grasping gestures, and improved grasping success rates. We emphasize that in the hand–object interaction, methods that enable high-quality grasping gesture generation for grasping objects are a fundamental step towards dexterous manipulation of powerful robots. The research of many scholars in this field and the improved methods provided in this paper will surely provide more reliable and higher-quality input data for subsequent research on task-specific constraint-oriented grasping optimization and simulation-to-reality migration techniques, as well as lay the foundation for building a more robust robot dexterous operation system. The structure of this paper is as follows: Section 2 primarily presents the improved contact map generation model and generation process in this paper’s grasping generation method, as well as the improved optimization strategies for grasping. Section 3 outlines the experimental setup and further demonstrates the generated visualization results. The efficacy of the proposed methodology is substantiated by a comparative analysis involving qualitative and quantitative evaluations, alongside a series of ablation experiments. Section 4 provides a comprehensive analysis and summary of the entire work.

## 2. Materials and Methods

### 2.1. Dataset and Hand Model

**Figure 2:**
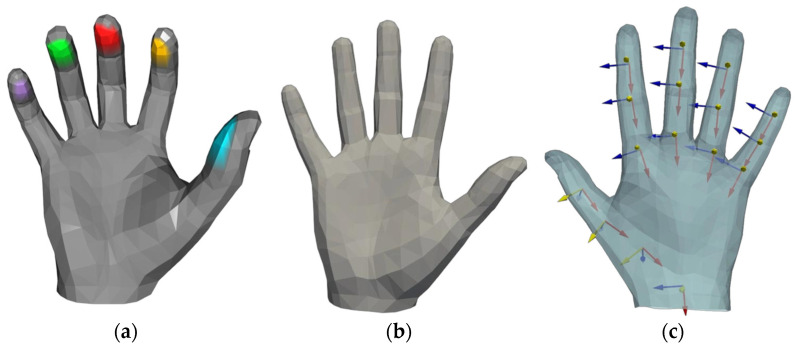
(**a**) Five different colors of the fingertips by manually labeling them for categorical differentiation. (**b**) Initial pose of the MANO hand model. (**c**) Adding a local coordinate system to the hand model while subsequent joint-angle solving is supervised.

This study uses the ObMan interactive dataset [4] for model training and grasping performance evaluation. As a large-scale benchmark dataset in the field of hand–object interaction synthetic images, ObMan integrates 2772 three-dimensional object meshes from eight typical functional categories in the ShapeNet [21] core database. Its rich object geometric shapes and diverse grasping configurations provide important support for grasping generation research. Unlike previous studies based on thermal contact mechanisms [5,13], we adopt the automatic annotation framework proposed in [16] to achieve precise contact modeling of the fingertip region. By analyzing the spatial topological relationships between hand and object point clouds, reliable contact map annotations are efficiently generated on the test set. This annotation method is based on the MANO hand model. As shown in Figure 2a, during contact map annotation, the fingertip regions are first manually circled using the Blender tool [22].

Additionally, for the regional segmentation of each finger component within the object point cloud, the points of the object are classified into regions corresponding to the thumb, index finger, middle finger, ring finger, little finger, and non-contact regions. It is important to note that when segmenting the object point cloud regions, since only the object point cloud is used, symbol distance (SDF) and alignment distance cannot be employed; only Euclidean distance can be utilized. Additionally, to avoid generating false contact maps or dividing overly small contact maps, the number of neighboring points K should not be too large or too small. In this study, we processed the object point cloud to retain 3000 points and set K = 50, which yielded good results.

To achieve subsequent grasping optimization and rationality in grasping generation, we need to accurately describe and utilize the MANO hand model, which parametrically represents hand morphology through shape parameters $\beta \in {\mathbb{R}}^{10}$ and pose parameters $\theta \in {\mathbb{R}}^{45}$. In the experimental setup, we keep the shape parameters *β* as the default zero vector to maintain the standard hand shape and control the overall spatial pose of the hand using rigid transformation parameters $t\in {\mathbb{R}}^{3}$ and rotation parameters R∈SO(3). It is worth noting that *θ* here is based on the PCA space pre-trained by the MANO model, and the pose parameters are constructed using a 45-dimensional (non-dimensional) representation that does not include wrist joint parameters, where the 45-dimensional parameters are the axis-intersecting representations of the joints of each finger [23]. Therefore, to achieve the joint angle constraint optimization mentioned in Section 2.3, we obtain the reference coordinate systems of each finger joint through anatomical annotation in the initial stage, as shown in Figure 2c, to achieve a differentiable transformation from joint axis angles to joint Euler angles, providing a quantitative basis for subsequent optimization. Additionally, for a more intuitive representation, we denote the five contact categories as c, using the symbols **T**, **H**, and **O** to represent the point cloud sets of the fingertip, entire hand, and object, respectively. We also divide the point cloud of each fingertip into regions denoted as **F**, which are used in the grasping optimization algorithm proposed in Section 2.3.

### 2.2. Generation of Detailed Contact Maps

Inspired by [12,24], we transfer common segmentation tasks to the generation of contact maps and improve the CVAE network architecture to propose a network architecture that combines CVAE with diffusion models and shares latent spaces (DiffCVAE).

The sharing and utilization of potential space in this network architecture acts in the training and generation phases. The training phase operates in a dual-threaded manner. The CVAE thread fuses object point cloud and contact map features, encodes them into latent variables, and captures the global structure of the contact maps. Concurrently, these latent variables are shared with the diffusion thread. The diffusion thread injects noise to perturb the latent variables and trains a noise predictor conditioned on the object geometric features, refining latent spatial details through diffusion loss minimization. During the generation phase, the CVAE encoder extracts geometric features from the object point cloud. Isotropic Gaussian noise is then sampled and reconstructed into latent variables via the reverse diffusion process, conditioned on the extracted geometric features. Finally, the decoder generates the fine-grained grasping contact maps.

The architecture predicts semantic labels for each fingertip and generates independent, detailed contact maps based on these predictions. We delineate the details of the training and generation phases in the subsequent sections.

**Figure 3:**
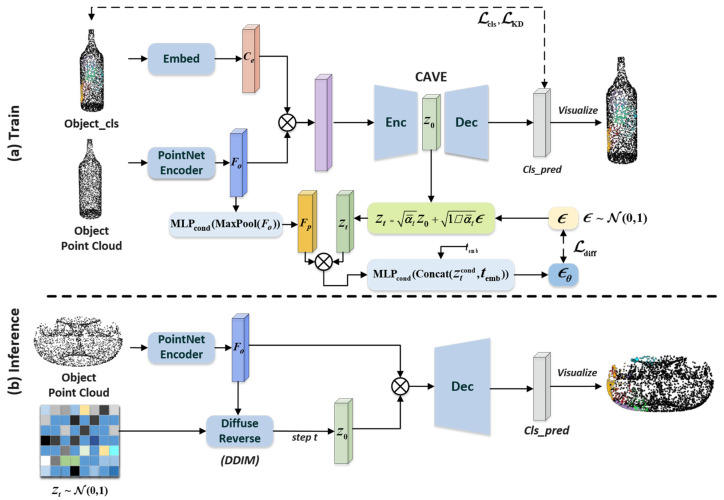
DiffCVAE architecture. (**a**) Inputs in training are the existing contact classification and object point cloud, and contact map generation and prediction of latent space noise are realized through the network of shared latent space. (**b**) Inputs in the generation phase are the object point cloud through iterative denoising of the latent space variables obtained from encoding, and contact map generation is obtained by the decoder.

Its network architecture takes the geometric point cloud and classification point cloud of the object as input and uses the decoder to predict and generate the grasp contact maps. The network architecture is shown in Figure 3.

During training, as shown in the top row of Figure 3, the CVAE network consists of an encoding stage and a decoding stage. In the feature extraction stage, to enable the generative network to comprehensively utilize the geometric and classification information of the object, we use the PointNet encoder [4] to extract the point cloud features $F_{o}\in {\mathbb{R}}^{256\times 576\times 3000}$ of the input object. Simultaneously, the input category obj_cls is mapped to the embedding vector space via an embedding layer, yielding the embedding category features $C_{\mathrm{emb}}\in {\mathbb{R}}^{256\times 64\times 3000}$ corresponding to the object contact maps, which serves as the conditional vector. Further, the point cloud features and conditional vector are concatenated in the second dimension, and the concatenated feature vector is input into the encoder. In the feature encoding stage, a feature compression channel is constructed using a multi-layer perceptron (MLP): first, higher-order features are extracted through stacked fully connected layers, and then, a max pooling layer is introduced to obtain a latent distribution $N\left(\mu ,\sigma^{2}\right)$ that conforms to a normal distribution.

In this network architecture, we improve the main CVAE network by embedding a learnable diffusion model module in the latent space. Specifically, in this model, a diffusion mechanism with random time step sampling is adopted: first, a random time step t∼μ(1,T) is generated that follows a uniform distribution; then, a noisy signal $\epsilon ∼N\left(0,1\right)$ is constructed at the *t*-th time step with the same dimension as the initial latent variable and diffusion forward propagation is performed to obtain the noisy latent variable $z_{t}\in {\mathbb{R}}^{256\times 1024}$, expressed as:

(1) $$z_{t}=\sqrt{{\bar{\alpha }}_{t}}z_{0}+\sqrt{1-{\bar{\alpha }}_{t}}\epsilon$$

where the noise rate is $\alpha_{t}=1-\beta_{t}$, and $\beta_{t}$ is a predefined noise scheduling parameter that increases with time step *t*, while ${\bar{\alpha }}_{t}$ represents the cumulative noise rate from time step 0 to time step *t*.

To establish conditionally constrained noise prediction, this module uses point cloud features as conditional inputs, first performs pooling and projects them into the latent space, and then injects conditional information into the latent variables. Note that addition is used here rather than feature concatenation to avoid feature dimension explosion. Since the diffusion model relies on time step information for temporal modeling, the time step embedding vector is concatenated in the second dimension as the input for noise prediction $N_{\mathrm{input}}\in {\mathbb{R}}^{256\times 1280}$. Finally, feature fusion is performed using a fully connected architecture based on a multi-layer perceptron (MLP) [25]. After normalization via a GroupNorm layer, a nonlinear transformation is completed using the SiLu activation function, ultimately outputting the predicted noise distribution $\epsilon_{\theta }$. This design incorporates feature dimensions while reasonably integrating conditional information and temporal features.

During the contact map generation stage, the unmodified CVAE encoding layer compresses the point cloud features of objects, leading to the loss of some key features. To obtain more refined latent variables, we reconstruct the initial latent variable $z_{0}$ through a reverse diffusion process [26]. First, based on the geometric dimensions of the object point cloud data (obj_pc), we generate a noise latent variable $z_{t}^{\prime }$ of corresponding size; then, using the DDIM (Denoising Diffusion Implicit Models) accelerated sampling mechanism, we perform reverse denoising iterations along the time steps, ultimately obtaining the refined latent space z as input to the CVAE decoder. Subsequently, we sample the latent space $z∼N\left(\mu ,\sigma^{2}\right)$ and replicate this latent variable n times along the channel dimension to expand it. The expanded latent tensor is concatenated with point cloud geometric features along the channel dimension to form a fusion feature matrix, and a contact map is generated via an MLP.

For the loss function in the training of the DiffCVAE model, we define three types of supervised loss functions. To enable the network to obtain a more detailed contact map from the input object point cloud, we define a reconstruction loss. Since we apply a segmentation task to predict multi-class contact maps, we set the reconstruction loss function $L_{\mathrm{cross}}$ to consist of a multi-class cross-entropy function $L_{\mathrm{cls}}$ and a multi-class dice loss $L_{\mathrm{dice}}$. Additionally, to encourage the encoder to learn a latent space that approximates a standard Gaussian distribution and to incorporate a latent space diffusion model, we define a regularization loss $L_{\mathrm{KD}}$, achieved by maximizing the KL-divergence [10]. Furthermore, we calculate the mean squared error between the predicted noise in the latent space and the generated noise fingertips as the diffusion loss, expressed as:

(2) $$L_{\mathrm{diff}}=E_{t,z_{0},\epsilon }\left[{\left\|\epsilon -\epsilon_{\theta }\right\|}^{2}\right]$$

An analysis of the CVAE network reveals that its latent space encodes the key features of high-dimensional input point clouds, directly influencing the decoding process. To this end, we introduce a diffusion loss to encourage the network to learn a more discriminative latent space from object point clouds. Overall, the complete loss function is formulated as:

(3) $$L=\lambda_{\mathrm{cross}}L_{\mathrm{cross}}+\lambda_{\mathrm{dice}}L_{\mathrm{dice}}+\lambda_{\mathrm{KD}}L_{\mathrm{KD}}+\lambda_{\mathrm{diff}}L_{\mathrm{diff}}$$

The corresponding weight losses are $\lambda_{\mathrm{cross}}=0.5$, $\lambda_{\mathrm{dice}}=0.9$, $\lambda_{\mathrm{KD}}=0.01$, and $\lambda_{\mathrm{diff}}=0.1$.

### 2.3. Proposed Grasping Optimization

To improve the realism of grasping with humanoid dexterous hands, we propose a physics-constrained and natural gestures consistency co-optimization framework (Physics-Naturalness Grasp Refinement, PNGR). Based on the 3D point cloud data of the target object and a detailed contact mapping map, we construct a multi-objective energy term that includes contact point distance constraints, normal constraints, grasp stability assessment, geometric penetration suppression, and joint pose angle optimization. This enables the generation of dexterous hand grasping poses that align with biomechanical characteristics and ergonomics. Effective contact and correct force application direction are prerequisites for grasping. To ensure actual contact between the hand and the object, a contact distance energy term $E_{\mathrm{con}}$ is introduced. This term guides the dexterous hand to the correct position by calculating the square of the minimum Euclidean distance between the fingertip points of fingers belonging to the same category and the contact mapping points. The energy term is expressed as:

(4) $$E_{\mathrm{con}}=\sum_{c=1}^{C}\sum_{i=1}^{N_{c}}E_{p}\left[\left(\min_{j}\|T_{i}^{c}-O_{j}^{c}{\|}_{2}^{2}\right)\right]$$

where $T_{i}^{c}$ is the coordinate of the i-th fingertip point of the c-class artificially defined, $O_{j}^{c}$ is the coordinate of the j-th point of the same-class finger generated by the improved CVAE model mentioned above, and $N_{c}$ is the number of fingertip points of the *c*-class finger. To ensure the correctness of the force application direction during grasping generation, inspired by [5], we introduce direction consistency energy [16], which is composed of tip direction consistency energy $E_{\mathrm{tip}}$ and finger direction consistency energy $E_{\mathrm{finger}}$, respectively represented as:

(5) $$E_{\mathrm{tip}}=\sum_{c=1}^{C}\sum_{i=1}^{\left|T^{c}\right|}E_{p}\left[{\left\|N(T_{i}^{c})-u(O_{k}^{c}-T_{i}^{c})\right\|}_{2}^{2}\right]$$

The $E_{\mathrm{tip}}$ energy term input consists of the surface normal vector $N(T_{i}^{c})$ of the points in the fingertip point cloud $T^{c}$ and the direction vector $\left(O_{k}^{c}-T_{i}^{c}\right)$ of the object contact point, where k is the index of the point in the contact point $O^{c}$ that is closest to $T_{i}^{c}$. This aligns the fingertip surface normal direction with the object contact point direction, ensuring that the force applied during grasping is perpendicular to the object surface.

(6) $$E_{\mathrm{finger}}=\sum_{c=1}^{C}\sum_{i=1}^{\left|F^{c}\right|}E_{p}\left[{\left\|N(F_{i}^{c})-u(O_{k}^{c}-F_{i}^{c})\right\|}_{2}^{2}\right]$$

The energy term $E_{\mathrm{finger}}$ is input as the surface normal vector $N(F_{i}^{c})$ of the finger point cloud $F^{c}$ and the direction vector of the object contact point $\left(O_{k}^{c}-F_{i}^{c}\right)$, where $F^{c}$ represents the finger point cloud set associated with category *c*, which is also manually defined, and $u\left(x\right)$ denotes the normalization operation. The optimization aims to align the direction of the entire finger with the surface direction of the object, thereby enhancing the coordination of the grasping action. To suppress local optima in the energy term, a randomization mechanism ($E_{p}\left(x\right)$) is introduced, where $E_{p}=(1-p)x+p\cdot 0$. This equation introduces a probability *p* to randomly discard some terms, increasing the randomness of the energy term optimization. Considering the differing effects of fingertip direction and finger direction on grasping stability, a weighted fusion is designed to achieve comprehensive optimization of the direction. The parameters in Equation (7) are set as follows: $\lambda_{\mathrm{tip}}=0.8$ and $\lambda_{\mathrm{finger}}=0.6$.

(7) $$E_{\mathrm{dir}}=\lambda_{\mathrm{tip}}E_{\mathrm{tip}}+\lambda_{\mathrm{finger}}E_{\mathrm{finger}}$$

Due to the highly non-convex nature of dexterous grasping optimization, to reduce the local optima caused by traditional optimization methods, we use the binary classification network [27] trained based on the PointNet architecture proposed in [16], taking the hand point cloud **H** and object point cloud **O** as inputs, to further predict the success of the grasping operation. To incorporate data-driven successful grasping patterns as prior knowledge into the traditional optimization framework, we use the binary cross-entropy loss function as an energy term, denoted as $E_{\mathrm{net}}$, for network supervision during the optimization process. This method addresses the local optima issue in non-convex optimization by leveraging the global guidance provided by the pre-trained network.

**Figure 4:**
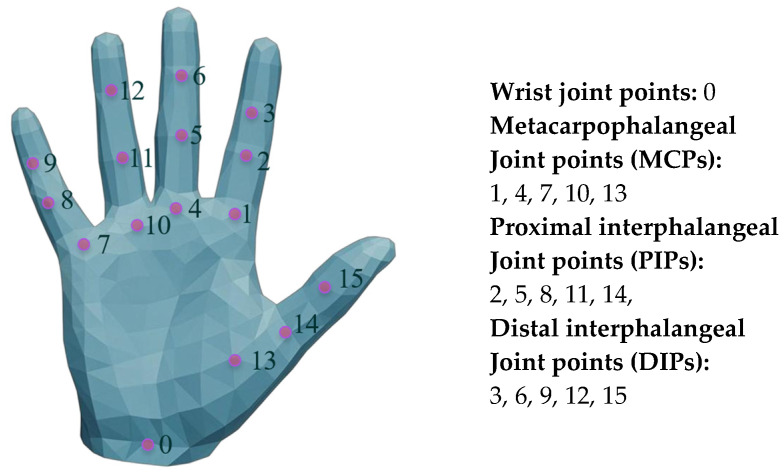
MANO hand model and index point labeling. Joint index annotation of hand models facilitates subsequent optimization algorithm optimization.

The addition of the above energy terms can already optimize basic grasping, but there are still issues with generating gestures that do not conform to natural anatomical gestures. To address this, we need to further introduce angle supervision energy terms ($E_{\mathrm{angle}}$). To calculate this energy term, we first refine the MANO hand model, mark its joints, and set the indices, as shown in Figure 4.

Analysis of the anatomical structure of the human hand and the motion characteristics of the joints of the humanoid manipulator shows that the metacarpophalangeal joint (MCP) has two modes of motion, namely, lateral swing and flexion–extension, while the proximal joint (PIP) and the distal joint (DIP) are only able to carry out the flexion–extension motion. Based on this, the corresponding angle constraints are set for each joint, and the specific parameters are shown in Table 1.

**Figure 5:**
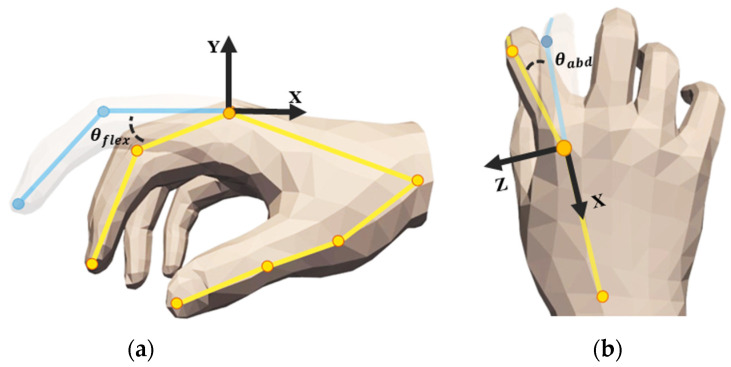
Metacarpophalangeal joint as an example; (**a**) flexion–extension angle; (**b**) lateral swing angle.

It is worth noting that the MANO model uses 45-dimensional pose parameters for hand characterization, and each parameter is presented as an axial angle of the joint angle, which needs to be converted to an Eulerian angle representation in order to achieve a detailed supervision of each joint $\theta_{ee}^{i}$, and the conversion expression is:

(8) $$\theta_{ee}^{i}=\left\{\begin{array}{l} \theta_{x}^{i}=\arctan2\left(R_{32}^{i},R_{33}^{i}\right)\\ \theta_{y}^{i}=\arctan2\left(-R_{31}^{i},\sqrt{{\left(R_{11}^{i}\right)}^{2}+{\left(R_{21}^{i}\right)}^{2}}\right)\\ \theta_{z}^{i}=\arctan2\left(R_{21}^{i},R_{11}^{i}\right) \end{array}\right.$$

(9) $$R^{i}={\left({\left(R_{p(i)}^{i}\right)}^{T}\cdot R_{\mathrm{tmp},p(i)}^{i}\right)}^{T}$$

where $R^{i}$ is the rotation matrix of the current pose of the hand joint concerning the template pose, as shown in Equation (9), where $R_{\mathrm{tmp},p(i)}^{i}$ is the local rotation matrix of the child joint concerning the parent joint, p(i) is the local rotation matrix of the template pose, and it is the parent node of the child joint *i*. Based on the obtained Euler angle matrix, one can further mention the side-swing angles $\theta_{\mathrm{abd}}$ of the MCP joints and the flexion and extension $\theta_{\mathrm{flex}}$ of the remaining joints, as shown in the Figure 5. From this, the angular loss [28] can be calculated and used as an angular supervised energy term for the optimization of the grasping gestures with the expression:

(10) $$L_{\mathrm{flex}}=\frac{1}{B\cdot N_{\mathrm{flex}}}\sum_{j=1}^{B}\sum_{i=1}^{N_{\mathrm{flex}}}\left[\mathrm{ReLU}{\left(\theta_{\mathrm{flex}}^{\min}[i]-\theta_{\mathrm{flex},j}^{i}\right)}^{2}+\mathrm{ReLU}{\left(\theta_{\mathrm{flex},j}^{i}-\theta_{\mathrm{flex}}^{\max}[i]\right)}^{2}\right]$$

(11) $$L_{\mathrm{abd}}=\frac{1}{B\cdot N_{\mathrm{abd}}}\sum_{j=1}^{B}\sum_{i=1}^{N_{\mathrm{abd}}}\left[\mathrm{ReLU}{\left(\theta_{\mathrm{abd}}^{\min}[i]-\theta_{\mathrm{abd},j}^{i}\right)}^{2}+\mathrm{ReLU}{\left(\theta_{\mathrm{abd},j}^{i}-\theta_{\mathrm{abd}}^{\max}[i]\right)}^{2}\right]$$

(12) $$E_{\mathrm{angle}}=L_{\mathrm{total}}=L_{\mathrm{flex}}+L_{\mathrm{abd}}$$

where *B* is the batch size set in the optimization, $L_{\mathrm{flex}}$ and $L_{\mathrm{abd}}$ are the joint losses, including the flexion–extension and side-swinging motion loss, and $N_{\mathrm{flex}}$ and $N_{abd}$ are the number of flexion–extension and side-swinging joints, respectively. To optimize the reasonable penetration between the fingertip and the object during grasping, penetration energy is introduced. Since the grasping generation is completely dependent on the point cloud, it is not possible to calculate the signed distance (SDF) [11] directly, so we use the method proposed in [10] to obtain the penetration energy $E_{\mathrm{pen}}$.

Based on obtaining each energy term, to balance the priority of different optimization objectives, guide the focus of the optimization process step by step, and generate a more natural and stable grasping gesture, we introduce an adjustable weight grasping strategy, and the total energy expression is:

(13) $$E=\mu_{c}\lambda_{\mathrm{dis}}E_{\mathrm{dis}}+(\mu_{s}-\mu_{c})\lambda_{\mathrm{dir}}E_{\mathrm{dir}}+\lambda_{\mathrm{net}}E_{\mathrm{net}}+\lambda_{\mathrm{pen}}E_{\mathrm{pen}}+\lambda_{\mathrm{angle}}E_{\mathrm{angle}}$$

where $\mu_{c}$ is the current iteration index in the optimization process and $\mu_{s}$ denotes the total number of iterations. These two parameters realize the adjustment strategy of weights. The parameters in Equation (13) are set as follows: $\mu_{s}=300$, $\lambda_{\mathrm{dis}}=0.5$, $\lambda_{\mathrm{dir}}=1.0$, $\lambda_{\mathrm{net}}=0.6$, $\lambda_{\mathrm{pen}}=10.0$, and $\lambda_{\mathrm{angle}}=10.0$.

## 3. Results

### 3.1. Experimental Platform and Configuration Parameters

Table 2 shows the configuration and parameters of the in-training optimization platform used in this study.

### 3.2. Quantitative Assessment of Contact Map Generation

**Figure 6:**
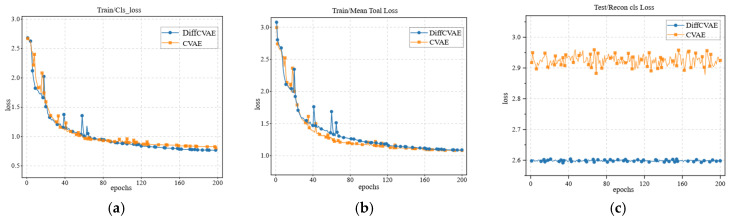
(**a**) shows the performance of the classification loss in the training of the base model and the improved model; (**b**) shows the performance on the overall loss; and (**c**) shows the performance of the model on the reconstructed classification loss on the test set.

Excellent grasping generation is predicated on fine contact maps. Therefore, we first quantitatively evaluate the generated network after the addition of the diffusion model against the original CVAE network, and we evaluate the effect of the diffusion model addition on the training loss by evaluating the effect of the diffusion model on the training loss on the training set, as shown in Figure 6a,b. The experimental results show that the inclusion of the diffusion model based on the shared potential space does not present a significant difference in the training loss curves of the CVAE generation module from the original model, while keeping the original network parameters and training conditions unchanged. Specifically, after 200 epochs of training cycles, the convergence trajectories of the two groups of models maintain similar levels with the final overall loss values as well as the reconstruction loss values. This phenomenon suggests that the introduction of the diffusion model has no substantial impact on the parameter optimization process of the CVAE generative network, further validating that this improved network allows for the integration of the diffusion model for the post-processing stage of generating samples rather than interfering with the core feature extraction and reconstruction mechanisms of the CVAE while maintaining the potential spatial sharing architecture. We validate the reconstruction loss of the two networks separately on the test set, and the results of the improved network on the test set are compared with the original network. As shown in Figure 6c, our improved DiffCVAE achieves a decrease in reconstruction loss, and the oscillation phenomenon is suppressed.

The above analysis shows that in the generation of contact maps, the shared diffusion model in the potential space has a better characterization ability for the accuracy and robustness of contact map generation. Based on this, we further evaluate and validate the grasping generation performance.

### 3.3. Quantitative Assessment of Grab Generation

We further quantitatively evaluate grasp generation by comparing it to typical grasp generation methods through three types of evaluation metrics: penetration volume, physical displacement, and grasp success rate.

Penetration Volume: We evaluate the penetration volume of all test samples. The hand–object grid was first voxelized at a voxel resolution of 0.5 cm [29], and the value of the penetration volume was obtained by calculating the intersection volume of the two grids [30]. This parameter is significant: a large penetration volume value implies that the hand model may cause physical damage to the surface of the object during the simulated grasping process, whereas a 0-penetration volume indicates that there is no substantial interactive contact between the hand and the object.

**Figure 7:**
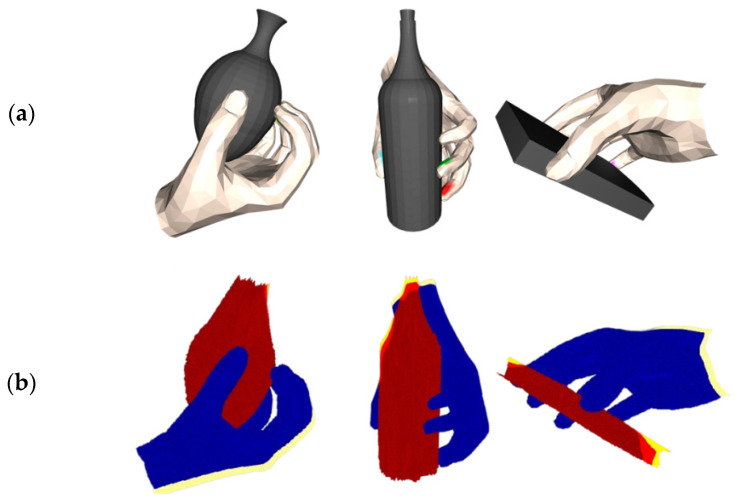
Simulated displacement visualization results in V-hacd. (**a**) displays algorithm-generated visualization of the grasp effect. (**b**) displays the virtual displacement visualization results of grabbing objects.

Physical displacement: This metric is a key measure of grasping stability. In this study, the generated hand model and the target grasping object are imported into the V-hacd physics simulator [31,32], which calculates the fingertip force that is positively correlated with the penetration volume and further acquires the displacement data of the grasped object’s center of mass within a specified time. The parameters of the simulation were set as follows: the gravitational acceleration was 9.8 m/s^2^; the friction coefficient between the object and the hand was the default value of 3.0; the time step was 1/240 s; the solver iterations were set to 150; and the simulator resolution was 1000. The visualization results are shown in Figure 7.

As shown, larger center-of-mass displacements intuitively reflect the presence of instability in the grasping process, which may lead to grasping failure, while smaller displacements indicate that the grasping system is in a relatively stable state.

Success rate: Reasonable and stable grasping should have a small penetration volume and simulated displacement at the same time, so we weigh these two key physical quantities to construct an evaluation system, and the specific criteria are set as follows: when the penetration volume is less than 5 cm^3^ and the simulated displacement is less than 2 cm, it is judged as a successful grasping. This dual threshold not only avoids damage to the surface of the object due to excessive penetration but also eliminates the risk of grasping failure due to large displacement, providing a feasible basis for the quantitative evaluation of grasping performance.

Naturalness Assessment: Prior work [10,33] assessed grasp naturalness by rendering grasps from three viewpoints and scoring them via a model [34]. To address potential inaccuracies from viewpoint limitations, we instead employ expert evaluation. Ten volunteers, all experts in dexterous hand design/development, hand rehabilitation R&D, or gesture recognition research, scored 15 randomly generated grasps presented in an interactive 3D viewer (allowing detailed inspection). The scores used a five-point scale with deductions for Non-biomechanical Pose: Violating human kinematics (Deduct: 0.5–1 pt); Finger Collisions: Inter-penetration (Deduct: 0.75 pt per colliding pair); and Abnormal Joint Angles: Unnatural rotations (Deduct: 0.5–1 pt). The final naturalness score for each grasp was calculated as 5 minus the total deductions incurred.

To exclude the interference of failed grabs on the evaluation of physical metrics, only successful grab samples (defined as penetration volume < 5 cm^3^ and simulated displacement < 2 cm) were included in this study, and the mean value of their penetration volume and physical displacement was calculated as the final evaluation value. The experimental results are summarized in Table 3.

Our method achieves improvements in both penetration volume and generation of grasping gestures, and the simulated displacements and grasping success rate maintain a good level. Since the optimization-only approach is based on the CVAE network in the base model GG for generating the contact maps, the optimization of the grasping gesture with the addition of joint supervision constraints results in an improvement of 3.27% in the grasping success rate and 2.15% in the naturalness of the grasping gesture, which suggests that the improved optimization approach contributes to better grasping. Using the improved contact map generation network-only approach, the lack of global optimization of the gesture spatial pose resulted in significantly poorer performance in penetration volume, simulated displacement, and naturalness, but no significant reduction in the grasping success rate due to finer contact map generation. In summary, the above demonstrates the synergy between contact map generation and gesture optimization, with the former providing accurate object contact position prediction and the latter ensuring the physical reasonableness of the gesture through joint motion constraints, which together form an optimization framework for grasping generation, resulting in a better performance of our approach.

### 3.4. Qualitative Assessment

**Figure 8:**
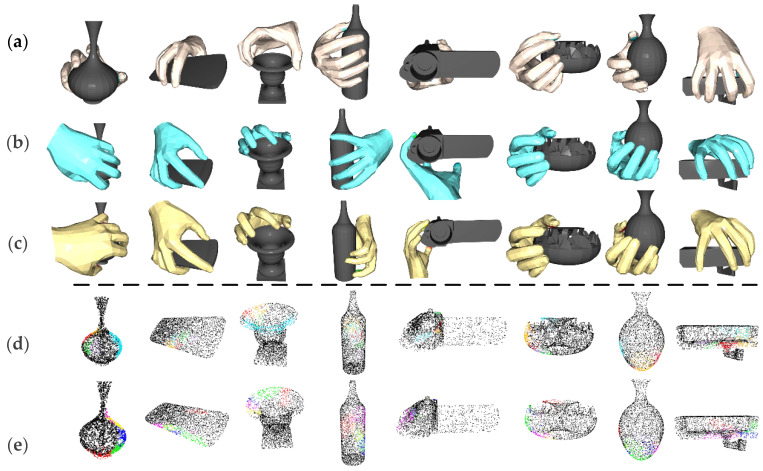
Qualitative experiment results. (**a**) shows the grasp generation results of our full method; (**b**) shows the grasp generation obtained by the optimization-only method; (**c**) shows the grasp generation results obtained by the base model; (**d**) shows the grasp contact maps obtained by utilizing the DiffCVAE generative network; (**e**) shows the grasp contact maps obtained by the original CVAE network.

The quantitative analysis intuitively compares the performance advantages of individual methods in terms of data, but for a more intuitive comparison, Figure 8 demonstrates the grasping generation effects of four methods: (1) the complete improvement method; (2) the optimization-only method (which employs GG’s contact map generation network); (3) the improvement-only network method (which generates contact maps based on DiffCVAE); and (4) the base GG method. The experimental results show that the optimization-only method has a similar grasping point distribution as the base method, due to following the contact map network of GG, and the improved network-only method exhibits a differentiated grasping contact point distribution, due to the use of DiffCVAE for generating the contact maps. To further demonstrate the advantages of the improved method in contact map generation, we compare three objects as examples.

**Figure 9:**
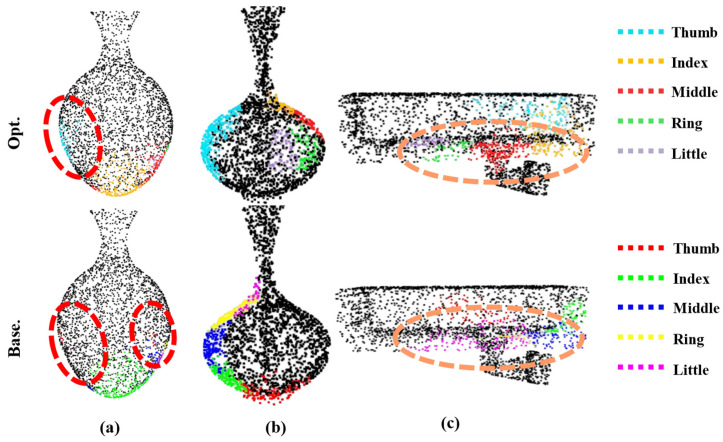
Comparison between the contact map generation effect of the improved model and the contact map generation effect of the base model. (**a**) Difference map generated from the contact graph during the jar grasping task; (**b**) Difference map generated from the contact graph during the vase grasping task; (**c**) Difference map generated from the contact graph during the camera grasping task.

First, it is essential to note that we use different colors for each fingertip index in the two types of methods to achieve a clear distinction. The comparison of Figure 9a clearly shows that the distribution of the thumb–fingertip contact region is more concentrated and reasonable in the contact maps generated by the improved model, whereas the contact maps generated by the base model exhibit the problem of dispersed and fine-grained thumb–fingertip contact regions. The comparison in Figure 9b further shows that in the grasping contact maps generated by the improved model, the grasping regions are uniformly distributed, and the contact regions between the fingers are concentrated and have clear boundaries. In contrast, the contact maps generated by the base model suffer from excessive concentration of grasping regions on one side and overlapping of contact regions, which will lead to grasping failure or finger collision. The comparison in Figure 8c also confirms the advantage of the improved model: for the fingertip contact regions of the middle finger, ring finger, and pinky finger, the contact maps generated by the improved model show a centralized distribution and are non-overlapping, while the contact maps generated by the base model show that the contact regions of the three fingers are fused, which is not conducive to generating reasonable grasping gestures.

**Figure 10:**
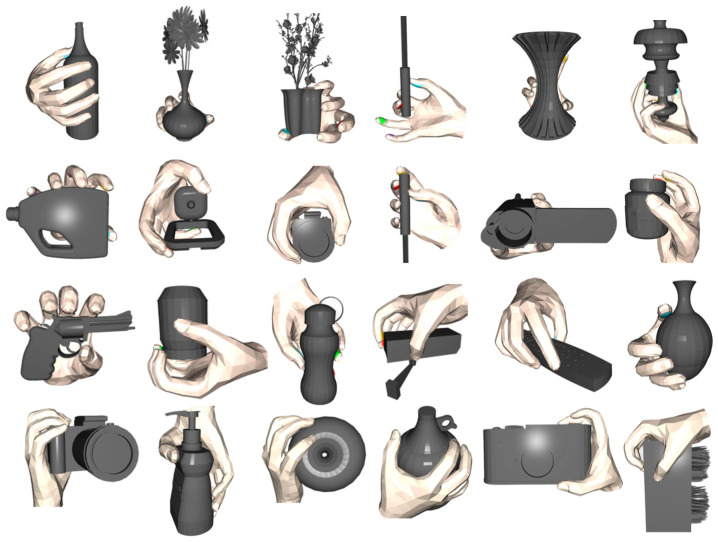
Visualization of grasp generation for different items.

In summary, our proposed improved method not only generates more accurate grasping contact points but also generates more ergonomically characterized hand postures. These findings confirm that the method makes a significant contribution to modeling grasping accuracy and grasp naturalness. Furthermore, to demonstrate the versatility of grasp generation, as shown in Figure 10, we tested grasp generation on a variety of objects and showcased the visualization results. For small objects, the generated grasping gestures make full use of finger dexterity to achieve stable grasping, and the generated gestures conform to the natural human grasping pattern. For complex and delicate objects, such as guns and screwdrivers, although the current method is not yet able to generate functional grasping gestures to meet their specific operational needs, it is still effective in generating gestures with reasonable contact areas and good grasping stability. For larger-sized objects, our method is also able to predict reasonable five-finger contact areas based on the contact map generation network, and it autonomously adjusts the hand posture through an optimization strategy that incorporates multiple types of energy terms to achieve stable grasping that includes palm contact. These results corroborate the effectiveness of the proposed method in hand–object interaction modeling and have significantly improved the stability and rationality of the generated gestures. Meanwhile, this study lays a solid theoretical and practical foundation for the subsequent exploration of task-oriented functional grasping research.

### 3.5. Ablation Experiments

The ablation experiments aim to quantitatively assess the specific contribution of each optimized energy term to the grasp generation algorithm, and the results of the experiments are shown in Table 4.

**Figure 11:**
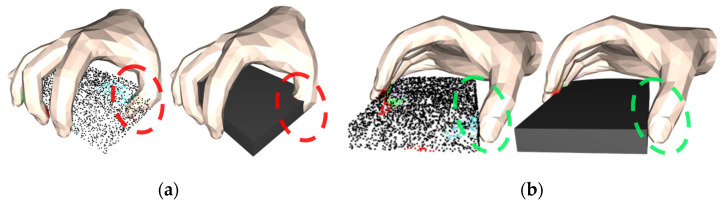
Positive visualization of $E_{pen}$ for comparison experiments. (**a**) is the capture effect image generated without adding $E_{pen}$, and (**b**) is the capture effect image generated with $E_{pen}$ added.

It is found that when the penetration energy term ($E_{pen}$) is removed, the grasping success rate will decrease by 65.3%, which directly leads to the loss of statistical significance in its comparison with other metrics. It should be emphasized that the core purpose of introducing the penetration energy term ($E_{pen}$) in the optimization process is to constrain the spatial topological relationship between the hand and the object, so as to avoid the generation of non-physical phenomena, such as fingers penetrating the object. Taking the tablet object grasping task as an example, Figure 11a presents the optimization result after removing $E_{pen}$, with the penetration phenomenon circled in red, which shows that the thumb severely penetrates the surface of the tablet, forming a typical ineffective grasping (this kind of situation may cause damage to the object in the real scenario); Figure 11b shows the optimization result with $E_{pen}$ retained, with the improved phenomenon highlighted in green, which has a significant effect on the suppression of the penetration phenomenon. Further analysis shows that removing $E_{pen}$ will cause the model to lose the ability to effectively constrain the penetration volume, which is a key indicator for determining the success rate of grasping, and thus, this operation will trigger a fundamental failure of the grasping mechanism.

And when the angular supervision energy term ($E_{angle}$) is masked, the lack of constraints on each finger joint leads to an increase in the penetration volume and simulated displacements, resulting in a 4.11% reduction in the success rate, reflecting the important role of joint angle constraints in grasping stability. $E_{net},$ on the other hand, learns reasonable hand–object interactions from a large number of datasets and thus improves the overall grasping generation quality, verifying the necessity of data-driven optimization. $E_{tip}$ and $E_{finger}$, on the other hand, optimize the grasping configurations at both the fingertip contact point and the overall finger contact direction levels, which together contribute to the improvement in the success rate. An analysis of the individual energy terms in the grasp generation optimization process reveals that removing the diffusion model from the grasp generation network increases the penetration volume by 7.03%, increases the simulated displacement by 7.69%, and reduces the grasp success rate by 0.83%. This result validates our overall design idea: combined with the comparison results of the reconstruction at loss one in the qualitative experiments, as shown in Figure 6c, it further indicates that the removal of the diffusion model reduces the generation accuracy of the contact map to a certain extent, which, in turn, affects the various evaluation indexes. The combination of complete energy terms (complete *E*) achieved the minimum volume error (1.85 cm^3^) and the optimal success rate (49.16%), demonstrating that each energy term has a complementary optimization role in grasp generation.

## 4. Discussion

The pros of this study include the following: We present a comprehensive analysis of our improved method. We utilize an improved contact map generation model, DiffCVAE, the core of which lies in the shared potential space design of the diffusion model and a CVAE, which enables the diffusion model to perform iterative denoising and refinement directly on the potential representations of the object features learned by the CVAE. It effectively utilizes the detail generation and correction capabilities of the diffusion model to overcome the feature loss problem that may exist in a single CVAE potential space, thus improving the quality of contact map generation. In the grasping optimization phase, a more natural and physically reasonable grasping is achieved through the consideration of physical constraints and the supervision of natural gestures. This method enhances the efficacy of grasping actions and the naturalness of the associated movement, while ensuring the simulated displacement is maintained within the simulation environment and minimizing the penetration volume. Furthermore, the grasp generation method utilizes object point cloud information exclusively, a development that renders future migration and deployment of dexterous hand grasping generation in robotics in real environments technically feasible.

The limitations of this study include the following: During the experimental process, it was observed that the optimization of gestures in the process of grasping generation requires multiple iterations to achieve the optimal result. This iteration necessitates a certain temporal cost, which cannot be met within the timeframe of grasping generation. We review the existing research. Zuo et al. proposed a GraspDiff grasping generation network [35], showing that the iterative denoising of the diffusion model, instead of the optimized iterative routine, can effectively reduce the time-consuming grasping generation. The method can be used for our subsequent research. The contact map generation network is trained only for similar items within classes and cannot obtain different contact positions for grasping and holding with diverse action intentions. To address this limitation, it is necessary to conduct research on cross-class grasping migration and contact map generation based on different intentions. [36].

## 5. Conclusions

In this study, we draw inspiration from existing hand–object interaction modeling generation methods and address their limitations. The goal is to generate physically plausible and highly humanoid grasping poses based on object point cloud information. While the actual task requires optimized grasping under functional constraints, academic consensus suggests that reliable synthesis of high-fidelity, stable, and naturalistic grasping modeling is a necessary foundation to support subsequent applications.

In order to enhance the quality of contact maps and generate more natural and physically reasonable grasp gestures, we propose a contact map generation method based on a conditional variational autoencoder (CVAE) and a diffusion model that shares a latent space (DiffCVAE). At its core, the diffusion model is iteratively optimized directly on the potential space of the CVAE to improve the fineness of the contact maps. And we propose an optimized grasp generation (grasp generation) framework (Diffangle-Grasp) that adheres to physical laws and natural gestures. The findings of the present study demonstrate that the proposed methodology exhibits superior performance in comparison to the conventional approach with regard to grasping stability, success rate, and the incorporation of natural gestures. This conclusion is substantiated by the outcomes of multi-class experiments. We believe that solving the fundamental challenge of generating grasps for high-quality hand–object interactions will provide technical feasibility for the subsequent practicalization of humanoid dexterous operating systems. However, it should be pointed out that the current optimization strategy has the problem of time-consuming grasping gesture iteration, and the grasp positions of different grasping actions in generic transfer scenarios also vary, requiring further research. Subsequent research will focus on algorithm efficiency and the exploration of migration learning technology, with the aim of overcoming the aforementioned technical bottlenecks. This will facilitate the further expansion of robot grasping technology to real-world application scenarios.

## Figures and Tables

**Table 1:** Angle constraints for each joint of the hand.

Angles	Index			Middle			Ring			Little			Thumb		
	1	2	3	4	5	6	10	11	12	7	8	9	13	14	15
$\theta_{abd}^{max}$	15°	0°	0°	5°	0°	0°	5°	0°	0°	0°	0°	0°	15°	0°	0°
$\theta_{abd}^{min}$	−5°	0°	0°	−5°	0°	0°	−15°	0°	0°	−15°	0°	0°	0	0°	0°
$\theta_{flex}^{max}$	90°	90°	90°	90°	90°	90°	90°	90°	90°	90°	90°	90°	90°	90°	90°
$\theta_{flex}^{min}$	0°	0	0	0°	0	0	0°	0	0	0°	0	0	0°	0	0

Here, $\theta_{abd}^{max}$ and $\theta_{abd}^{min}$ represent the maximum and minimum values of the lateral swing angle of the joint. In contrast, $\theta_{flex}^{max}$ and $\theta_{flex}^{min}$ represent the maximum and minimum values of the flexion and extension angles of the joint.

**Table 2:** Experimental flat configuration and parameters.

Parameter	Configuration
CPU	Intel(R) Xeon(R) Platinum 8457C
GPU	L20
GPU Video Memory	48 GB
Operating System	Ubuntu 22.04
Training Environment	PyTorch 2.0.1 + Pytorch3d 0.77 + Python 3.10 + CUDA 11.8

**Table 3:** Comparative experiments.

Methods	Volume (cm^3^)	Displacement (cm)	Succ. Rate (%)	Ntrl. Scores
GG(complete) [16]	2.08	0.76	46.98	3.64
GG(only opt.) [16]	1.98	0.84	48.75	3.71
GT [4]	2.20	**0.75**	**52.28**	3.51
FG [8]	1.93	0.79	48.27	3.68
GF [33]	2.38	0.89	20.60	2.93
GA [10]	3.65	0.80	35.61	3.43
Ours (only opt.)	2.05	0.81	48.57	3.76
Ours (only Diff.)	2.17	0.84	47.14	3.40
Ours (complete)	**1.85**	0.78	49.16	**3.72**

The bolded data in the table represents the best performance in the comparative experiments.

**Table 4:** Ablation experiment.

Energy	Volume (cm^3^)	Disp. (cm)	Succ. R. (%)
w/o $E_{tip}$	2.67	0.86	44.58
w/o $E_{finger}$	2.56	0.78	45.88
w/o $E_{net}$	2.68	0.82	42.86
w/o $E_{pen}$	3.86	1.21	17.07
w/o $E_{angle}$	2.17	0.84	47.14
w/o Diff.	1.98	0.84	48.75
complete *E*	**1.85**	**0.78**	**49.16**

The bolded data in the table represents the best performance in the comparative experiments.

## Data Availability

The datasets presented in this paper are open source. The ObMan dataset can be downloaded at https://www.di.ens.fr/willow/research/obman/data/ (accessed on 15 July 2025), and the ShapeNet dataset can be downloaded at https://shapenet.org/ (accessed on 15 July 2025). If you need the original training code, please contact zzh951120@gmail.com.
